# Cancer Testis Antigen, NOL4, Is an Immunogenic Antigen Specifically Expressed in Small-Cell Lung Cancer

**DOI:** 10.3390/curroncol28030179

**Published:** 2021-05-20

**Authors:** Ye-Rin Kim, Ki-Uk Kim, Jung-Hee Lee, Deok-Won Kim, Jae-Heun Chung, Yeong-Dae Kim, Dong-Hoon Shin, Min-Ki Lee, Yong-Il Shin, Sang-Yull Lee

**Affiliations:** 1 Department of Biochemistry, School of Medicine, Pusan National University, Beomeo-ri, Mulgeum-eup, Yangsan 50612, Korea; gbigtree@naver.com (Y.-R.K.); haga88@naver.com (D.-W.K.); 2 Department of Internal Medicine, Pusan National University Hospital, 1-10 Ami-dong, Seo-gu, Busan 49241, Korea; uk303@hanmail.net (K.-U.K.); leemk@pusan.ac.kr (M.-K.L.); 3 Department of Pathology, School of Medicine, Pusan National University, Beomeo-ri, Mulgeum-eup, Yangsan 50612, Korea; 4unii@hanmail.net (J.-H.L.); donghshin@chol.com (D.-H.S.); 4 Department of Internal Medicine, Pusan National University Yangsan Hospital, Yangsan 50612, Korea; jhchung7942@gmail.com; 5 Department of Thoracic Surgery, Pusan National University Hospital, 1-10 Ami-dong, Seo-gu, Busan 49241, Korea; domini@pusan.ac.kr; 6 Research Institute for Convergence of Biomedical Science and Technology, Pusan National University Yangsan Hospital, Yangsan 50612, Korea; rmshin@pusan.ac.kr

**Keywords:** KP-SCLC-29, NOL4, cancer/testis antigen, small-cell lung cancer, SEREX

## Abstract

To identify cancer/testis (CT) antigens and immunogenic proteins, immunoscreening of testicular and small-cell lung cancer cell line NCI-H889 cDNA libraries was performed using serum obtained from a small-cell lung cancer (SCLC) patient. We obtained 113 positive cDNA clones comprised of 74 different genes, designated KP-SCLC-1 through KP-SCLC-74. Of these genes, 59 genes were found to be related to cancers by EMBASE analysis. Three of these antigens, including KP-SCLC-29 (NOL4), KP-SCLC-59 (CCDC83), and KP-SCLC-69 (KIF20B), were CT antigens. RT-PCR and western blot analysis showed that NOL4 was frequently present in small-cell lung cancer cell lines (8/9, 8/9). In addition, NOL4 mRNA was weakly, or at a low frequency, or not detected in various cancer cell lines. Our results reveal that NOL4 was expressed at protein levels in small-cell lung cancer tissues (10/10) but not detected in lung adenocarcinoma and squamous cell carcinoma by immunohistochemical analysis. Serological response to NOL4 was also evaluated by western blot assay using NOL4 recombinant protein. A humoral response against NOL4 proteins was detected in 75% (33/44) of small-cell lung cancer patients and in 65% (13/20) of healthy donors by a serological western blot assay. These data suggest that NOL4 is a specific target that may be useful for diagnosis and immunotherapy in SCLC.

## 1. Introduction

Globally, small-cell lung cancer (SCLC) accounts for 13–15% of all lung cancers, with about 250,000 diagnosed annually, and is a malignant tumor with a very high cancer-related mortality rate and a 5-year survival rate of 1–5% [1]. Only two stages of SCLC are distinguished clinically: limited disease (LD) with tumor confined to one hemithorax only versus extensive disease (ED) with metastases in the contralateral chest or at distant sites [2]. For 30 years, treatment of SCLC has not changed in nature, and effective treatment options for the recurrent disease have been lacking [2]. There are significant barriers to progress in SCLC because of small diagnostic biopsies and the rare use of surgical resection in standard treatment, and rapid disease progression [2]. However, for the past five years, the development of clinical therapeutic strategies, including immunotherapy and new molecular targets, has been undertaken at the molecular level through the discovery of molecular mechanisms and development of models for SCLC [3,4,5]. Therefore, the discovery of molecular targets for early detection and taking a new approach to therapy in SCLC are important. 

The immunotherapy approach to SCLC has been studied, including vaccine studies, interferon-α, and several clinical trials of immune checkpoint inhibitors [5]. Recent progress in tumor immunology based on the molecular identification of tumor antigens may allow the combination of early detection and immunotherapy as a promising treatment for several cancers [6]. However, compared to other solid tumors, there is little knowledge on tumor antigens in SCLC. 

The repertoire of tumor antigens recognized by the immune system is referred to as the cancer immunome [7]. The immunome comprises antigens defined by T-cell epitope cloning [8], major histocompatibility complex (MHC) peptide elution [9,10], and serological expression cloning [11,12]. Among these approaches, the serological analysis of recombinant cDNA expression libraries (SEREX) approach is based on the screening of cDNA expression libraries generated from tumor tissues of various origin or cancer cell lines [12]. SEREX is an effective method to identify the tumor antigens and has been applied to a wide range of tumor types [13,14].

One of the categories of tumor antigens has been referred to as cancer/testis (CT) antigens. Cancer/testis (CT) antigen is a group characterized by specific expression patterns restricted to tumors and germ cells, with more than 200 genes found to belong to this group [15]. Some CT antigens such as MAGE-A3 and NY-ESO-1 have been evaluated for their clinical therapeutic effect [16,17,18]. Additionally, CT antigens such as SPAG9 have been studied for their potential as biomarkers for diagnosis and prognosis [19,20]. Although many CT antigens have been studied, their function, particularly the role of these genes in gametogenesis and carcinogenesis, is not fully understood. However, evidence continues to accumulate indicating that CT antigens, including NY-SAR-35, CT45A, SSX, and CAGE, are probably involved in cell cycle regulation, cell survival, apoptosis, and metastasis [21,22,23,24]. These findings support the usefulness of CT antigens to develop more effective cancer vaccines [25]. In the present study, we performed the SEREX method to study further defining the spectrum of immunogenic proteins in SCLC and identified 74 antigens, including three CT antigens: NOL4, CCDC83, and KIF20B. A specific focus was given to NOL4 gene to determine its potential as a possible CT antigen in SCLC.

## 2. Materials and Methods

### 2.1. Biospecimens and Cell Lines

The biospecimens and data used for this study were provided by Biobank of Pusan National University Hospital (a member of the Korea Biobank Network), the Institutional Biobank Project, and the Department of Pathology and Internal medicine of Pusan National University Hospital after diagnosis. Nine SCLC cell lines (NCI-H69, NCI-H82, NCI-H146, NCI-H187, NCI-H378, NCI-H889, NCI-H1688, DMS53, and SW1271) were obtained from the cell repository at the Memorial Sloan-Kettering Cancer Center and the American Type Culture Collection (ATCC). NCI-H69, NCI-H82, NCI-H146, NCI-H187, NCI-H378, NCI-H889, and NCI-H1688 were maintained in RPMI-1640 medium (Welgene Inc., Deagu, Korea), and DMS53 and SW1271 were maintained in Waymouth’s medium (Thermo Scientific, Grand Island, NY, USA) and Leibovitz’s L-15 medium (ATCC), respectively. All these media were supplemented with 10% fetal bovine serum, 2 mM l-glutamine, 100 U/mL penicillin, and 100 μg/mL streptomycin. 

### 2.2. Total RNA Extraction from Tissues and Cell Lines

Total RNA from human normal tissue was purchased from Clontech Laboratories (Mountain View, CA, USA), Inc. Total RNA of various cancer cell lines was obtained from a reservoir previously used in our laboratory or extracted using TRIzol reagent (Invitrogen, Carlsbad, CA, USA), and cDNA was synthesized using reverse transcriptase (Promega, Madison, WA, USA).

### 2.3. Preparation of cDNA Library and Serum

Poly(A)+ RNA (5 μg) from NCI-H889 and normal testis was used to construct a cDNA library, following the manufacturer’s instructions of the SMART cDNA library construction kit (Clontech Inc., Mountain View, CA, USA). The library contained approximately 1 million recombinants and was used for immunoscreening without prior amplification. Serum from one patient who had been diagnosed with extensive SCLC and survived at least 50 months was used for immunoscreening.

### 2.4. Preparation of Human Sera

To remove antibodies that react with non-specific antigens, the serum was absorbed with *E. coli*/bacteriophage lysates as described previously [26]. First, wild-type λ ZAP Express bacteriophage was incubated with E. coli XL1 Blue MRF’ at 37 ℃ 15 min to allow the phage to attach to the cells. Then, phage and *E.coli* were evenly plated on NZY/1.5% agar petri-dish with NZY Top agarose and incubated overnight at 37 ℃. Next day, after confirming that plaque was formed, the coupling buffer (0.5M NaCl + 0.1M NaHCO_3_) was added to the plates and stirred overnight at 4 ℃. The resulting supernatant was collected, and residual *E. coli* was dissolved by ultrasonic waves. After that, the lysate was bound to CNBr-Sepharose 4B bead. The sera were incubated with beads in a rotor-wheel at 4 ℃ overnight. The next day, the sera supernatant was transferred from the bead sediment to a new tube, diluted 1:200 with 0.2% NFDM, and 0.02% NaN_3_ and 0.05 mg/mL of gentamicin were added.

### 2.5. Immunoscreening

Immunoscreening of the cDNA library was performed as described previously [27]. Briefly, the recombinant phage constituting the cDNA library was transfected into *E. coli* MRF cells and plated at a concentration of 5000 pfu/dish. The plate was incubated at 37 °C for 8 h and then transferred to nitrocellulose filters (Schleicher & Schuell, Keene, NH, USA). The filters are then reacted with the patient’s serum. After that, the filter reacts with the AP-conjugated secondary antibody, and finally, the serum-reactive clone is detected through the reaction with BCIP/NBT. After screening, the positive clones were extracted from the plate and finally sequenced commercially (Macrogen, Seoul, Korea).

### 2.6. Reverse Transcriptase-PCR (RT-PCR) Analysis

The cDNA preparations used as templates for RT-PCR reactions were prepared using 1 μg of total RNA using M-MLV reverse transcriptase (Promega, Madison, WA, USA). PCR primers were as follows: NOL4 forward: 5′-AAACGGGGCCAAATGGAGAA-3′, reverse; 5′-TTGAACTGTGCCCCAGAGTC-3’ (59 ℃), GAPDH forward; 5′-CCACCCATGGCAAATTCCATGGCA-3′, reverse; 5′-TCTAGACGGCAGGTCAGGTCCACC-3′ (58 ℃). The cDNA templates used were normalized on the base amplification of GAPDH. For PCR, 20 μL reaction mixtures were utilized, consisting of 1 μL cDNA, 0.5 μM gene specific forward and reverse primers, and 2× TOPsimpleTM DyeMIX-Tenuto (Enzynomics, Daejeon, Korea). Reaction mixes were heated to 95 ℃ for 5 min, followed by 30 cycles of 95 ℃ for 30 s, 59 ℃ for 30 s, and 72 ℃ for 1 min (final cycle: 72 ℃ for 5 min). Amplified products were analyzed on 1.5% Agarose/EcoDyeTM Nucleic Acid Staining Solution (Biofact, Seoul, Korea) gels.

### 2.7. Western Blot Analysis

Cell lysates were separated by 10% sodium dodecyl sulfate-polyacrylamide gel electrophoresis (SDS-PAGE) followed by being transferred onto nitrocellulose membranes. The primary antibodies were NOL4 antibody (1:2000 dilution, Cat.# H00008715-M01, Novus, Southpark Way, CO, USA) and anti-β actin antibody (Sigma-Aldrich, St. Louis, MO, USA, 1:5000 dilution). Membranes were incubated with horseradish peroxidase (HRP)-conjugated goat anti-mouse-IgG secondary antibody (Enzo Life Science Inc., Farmingdale, NY, USA, 1:5000 dilution) and detected by enhanced chemiluminescence reagent (Perkin-Elmer Life Science, Waltham, MA, USA).

### 2.8. Serological Assay

A 100 ng of NOL4 protein (Origene Technology Inc., Rockville, MD, USA, Cat.#TP322286) were separated by sodium dodecyl sulfate-polyacrylamide gel electrophoresis followed by being transferred onto nitrocellulose membranes. After blocking with TBST (TBS and 0.1% Tween-20) containing 5% skim milk for 1 h at room temperature, the membranes were incubated in each patient’s sera (1:200 dilution) overnight at room temperature. The membranes were washed and incubated with HRP-conjugated sheep anti-human IgG antibody (GE Healthcare) (1:5000 dilution) for 1 h at room temperature. After washing with TBST and incubating with ECL reagent (PerkinElmer, Waltham, MA, USA), the membranes were exposed to Kodak medical X-ray film. A total of 20 samples of healthy donor sera and a total of 44 samples of SCLC patients were used for this analysis. Of the patient sera samples, 13 samples were from patients diagnosed with limited disease and 31 were from patients diagnosed with extensive disease.

### 2.9. Immunohistochemistry

Sections from formalin fixed paraffin-embedded blocks diagnosed at the Department of Pathology, Yangsan Pusan National University Hospital were used. Staining was conducted with the peroxidase-based EnVision Detection kit (DakoCytomation, Carpinteria, CA, USA) by following the user manual. Briefly, tissue sections were deparaffinized and hydrated in xylene and graded alcohol series. Then, the sections were incubated with the NOL4 antibody (Cat.# H00008715-M01, Novus, Southpark Way, CO, USA) for 1 h at room temperature. After incubation with EnVision solution, the 3,3′-diaminobenzidine (DAB) substrate was added followed by hematoxylin counterstaining and mounting.

## 3. Results

### 3.1. Identification and Characterization of SCLC Antigens by SEREX

To evaluate the expression frequency of CT antigens in SCLC cell lines, six SCLC cell lines above were co-typed for expression of LAGE-1, MAGE-1, MAGE-3 MAGE-4, MAGE-10, CT-7, NY-ESO-1, NY-SAR-35, SSX1, SSX2, SSX4, KP-LU-35, SCP-1, and NY-TLU-57 by RT-PCR. Among the six cell lines, NCI-H889, which expressed 11 of the 14 CT antigen transcripts (data not showed) as well as normal testis, was chosen as the cDNA expression library sources for SEREX. Immunoscreening of the NCI-H889 SCLC cell line and testicular cDNA expression libraries with selected serum resulted in the isolation of 113 seroreactive cDNA clones. These clones included 74 antigens and designated KP-SCLC-1 to SCLC-74 (Table 1). 

Seventy-four isolated genes were analyzed using bioinformatics tools including the GeneCards database (http://bioinfo.weizmann.ac.il/cards-bin/) (accessed on 15 November 2020) and EMBASE (https://www.embase.com/) (accessed on 15 November 2020). Especially, we investigated whether there was at least one published research paper in which these genes were related to cancer. Of the 74 genes, 80% were found to be related to cancer (Table 1). The 74 antigens were functionally identified as known and predicted proteins. The antigens were categorized into several functional group, for example, oncogenes/tumor suppressor genes, DNA binding/transcription related gens, RNA processing/translation related genes, regulatory/signal transduction related genes, metabolism related genes, and others, which included autoantigens and CT antigens. Of these functional groups, we found interesting genes, including oncogenes/tumor suppressor genes (KP-SCLC-2, -4, -7, -17, -19, and -74), autoantigens (KP-SCLC-1, -15, and -58), and specifically CT antigens (KP-SCLC-29, -59, and -69). KP-SCLC-29, as known nucleolar protein 4 (NOL4), was previously reported as CT 125 [28,29]. KP-SCLC-59 was reported as CT 148 and has been identified as KP-CoT-23 (CCDC83) in colon cancer [27]. Additionally, KP-SCLC-69 was reported as CT90 and was known as KIF20B with carcinogenic function [30].

### 3.2. The mRNA and Protein Expression Profiles of NOL4

**Figure 1:**
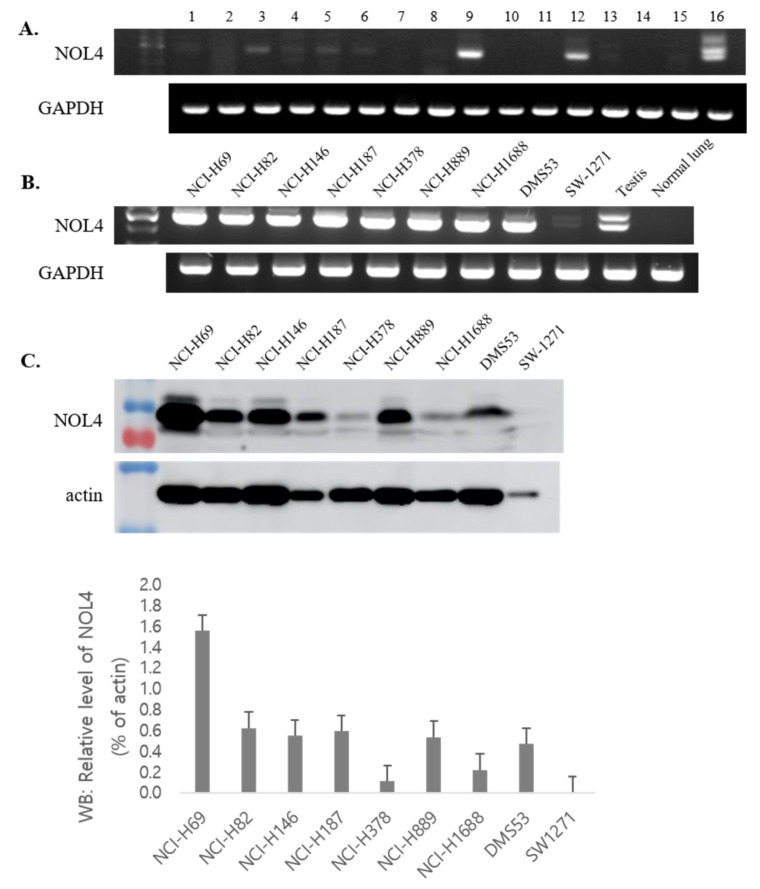
The mRNA and protein expression of NOL4 in normal tissues and small-cell lung cancer (SCLC) cell lines. RT-PCR was performed on mRNA expression of NOL4 in normal tissues (**A**) and SCLC lines (**B**). Western blot analysis was performed to evaluate the protein expression of NOL4 in SCLC cell lines, and the intensity ratio of each band was analyzed by Image J (**C**). Normal tissues were indicated as follows: 1, spleen; 2, thymus; 3, prostate; 4, ovary; 5, small intestine; 6, colon; 7, leukocyte; 8, heart; 9, brain; 10, placenta; 11, lung; 12, pancreas; 13, liver; 14, skeletal muscle; 15, kidney; and 16, testis. The cDNA templates were normalized using GAPDH, as shown in the bottom panel.

**Figure 2:**
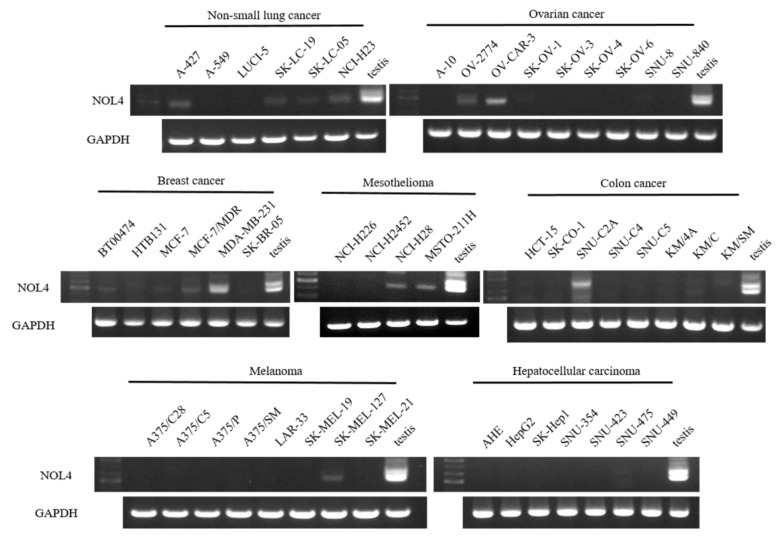
The mRNA expression of NOL4 in several cancer cell lines.

To investigate the restricted expression of NOL4 mRNAs in normal adult tissues, RT-PCR was performed. As shown in Figure 1A, NOL4 mRNA was strongly expressed in testis and showed high expression in brain and pancreas, and there was little expression in prostate, small intestine, brain, and pancreas. NOL4 mRNA and proteins were frequently present in SCLC cell lines (8/9, 8/9), respectively (Figure 1B,C). In addition, NOL4 mRNA was expressed weakly, detected at a low frequency, or not detected, in non-small-cell lung cancer, ovarian cancer, breast cancer, mesothelioma, colon cancer, melanoma, and hepatocellular carcinoma (Figure 2). These results indicate that NOL4 may be cancer/testis antigen that is frequently expressed in SCLC.

### 3.3. NOL4 Protein Is Specifically Expressed in Tissues of SCLC

**Figure 3:**
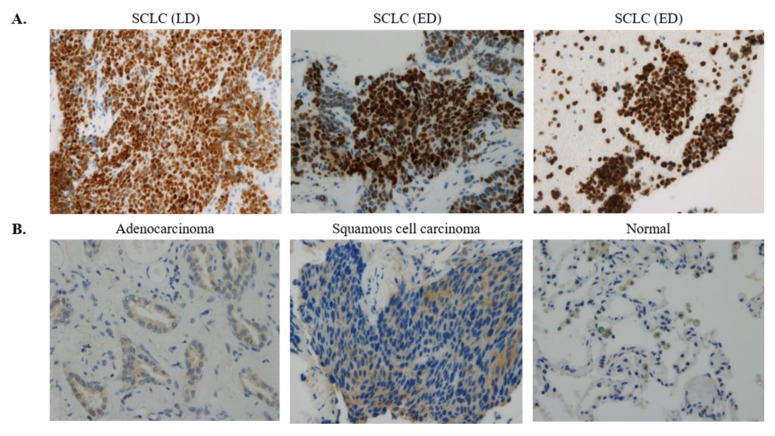
Immunohistochemical staining of NOL4 proteins. Panels refer to representative sections showing positive staining (**A**) and negative staining (**B**). Original magnification is 100×.

The protein expression of NOL4 was examined in SCLC and non-SCLC by immunohistochemical analysis. Figure 3A,B shows a representative picture of SCLC tissues, in which brown nucleus staining of NOL4 is visible in tumor cells, and pictures of negative control. NOL4 was expressed at protein levels in 10/10 of SCLC tissue specimen but not detected in lung adenocarcinoma and squamous cell carcinoma. We are analyzing NOL4 protein expression in more tissue samples from SCLC and non-SCLC patients (see discussion).

### 3.4. Seroreactivity of NOL4 by Western Blot Analysis

To determine whether immune recognition of the NOL4 proteins is cancer-related, allogeneic sera samples obtained from 44 patients with SCLC and 20 normal individuals were tested for NOL4 reactivity by western blot analysis. As shown in Table 2 and Supplementary Materials Figure S1, humoral response against NOL4 protein was detected in 75% (33/44) of SCLC patients, and, more specifically, 77% (24/31) of extensive-disease-SCLC patients and 69% (9/13) of limited-disease-SCLC patients, respectively. On the other hand, it was found in 65% (13/20) of healthy patients. The correlation between NOL4 expression and positive IgG was not evaluated due to a lack of paired samples; therefore, this requires further investigation in a future study.

## 4. Discussion

An attempt to identify tumor antigens in SCLC was undertaken by Güre et al [31]. They isolated 14 genes from two SCLC cell lines using pooled sera of SCLC patients. To identify additional immunoreactive antigens in SCLC patient, we performed SEREX analysis and isolated 74 different genes, designated KP-SCLC-1 through SCLC-74. The 74 antigens identified in this SEREX analysis of SCLC represent a broad spectrum of cellular components. A striking feature of these antigens is the diversity of genes recognized by the serum of patient with SCLC, as well as 80% genes associated with cancer (Table 1). Among these isolated antigens, we found 3 previously defined CT antigens, including KP-SCLC-29 (NOL4), KP-SCLC-59 (CCDC83), and KP-SCLC-69 (KIF20B).

NOL4, known as nucleolar protein 4, was identified a novel methylated tumor suppressor gene in head and neck cancer and cervical cancer [29,32]. Additionally, NOL4 gene is a biomarker candidate of many CT antigens for diagnosis and prognosis of prostate cancer [33] and is a new potential therapeutic target in glioblastoma stem cells [34]. In our study, NOL4 was isolated for the first time from SCLC patients by SEREX. NOL4 mRNA and protein were highly and specifically expressed in SCLC cell lines and tissue sections. These results suggest that NOL4 may be a biomarker candidate for SCLC prognosis and diagnosis. To address this possibility, our collaborators are examining NOL4 protein expression in more tissue samples from SCLC patients and investigating whether NOL4 expression is correlated with the histological grade and clinical stage of tumors. They found a high and specific expression of NOL4 in more tissue blocks and micro tissue array of SCLC patients, and they are currently analyzing the results based on clinical history of SCLC patients (personal communication with Dr. Dong-Hoon Shin and Dr. Jung-Hee Lee, who are working at the Department of Pathology, Yangsan Pusan National University Hospital).

In addition, anti-NOL4 antibody was detected at high frequency in SCLC sera samples by western blot analysis. The correlation between NOL4 expression and anti NOL4 IgG was not evaluated due to a lack of paired samples; therefore, this should be investigated in a future study. Nonetheless, NOL4 recognition by sera from SCLC patients and healthy individuals indicates that NOL4 is an immunogenic CT antigen. The significant frequency of IgG antibodies responses to NOL4 suggested that further investigation is required to evaluate immune response from this antigen.

We subsequently found KIF20B gene, which was previously reported as an oncogenic CT antigen in bladder cancer [30]. KIF20B, which is known as MPHOSPH1, one of the kinesin superfamily proteins and one of the most promising oncogenic targets, has been reported to play an essential role in the carcinogenesis and progression of several kinds of cancers, including bladder cancer, breast cancer, renal cancers, and hepatocellular carcinoma [35]. Clinical trial of peptides cancer vaccine therapy, which was derived from KIF20B, showed sufficient tolerance and effective induction of peptide-specific cytotoxic T lymphocytes [36,37].

CCDC83 was previously reported as a CT antigen, KP-CoT-23, from colon cancer by SEREX [27]. KP-CoT-23a and b genes were frequently expressed in several tumor types and cancer cell lines, especially in colon cancer. In SCLC, KIF20B and CCDC83 may need to be studied further.

In conclusion, we reported for the first time the isolation of NOL4 antigen from SCLC patient serum by SEREX. We demonstrated high NOL4 mRNA and protein expression and high seroreactivity, which may have a favorable impact on diagnosis and immunotherapy of SCLC patients. SEREX-derived CT antigens have been shown to induce CD8+ CTLs [38,39], and a positive correlation was observed between serum positivity for IgG antibody and induction of CD8+ CTLs against the cancer testis antigen NY-ESO-1 [39]. The significant frequency of IgG antibody responses against NOL4 suggested that the strong immunogenicity and CD4 and CD8 T-cell responses against the antigens should be investigated. For the application of the NOL4 in immunotherapy and cancer diagnosis, more detailed studies such as epitopes purification, antibody synthesis, and immunohistochemical analysis are required.

## Figures and Tables

**Table 1:** Antigens identified by SEREX in SCLC patient.

KP-SCLC-Antigen ^a^	Gene Name/REFSEQ mRNAs	Cancers-Related Published Papers ^b^	KP-SCLC-Antigen	Gene Name/REFSEQ mRNAs	Cancers-Related Published Papers
1	EIF5B/NM_015904.4	Yes	38	CTBP2/NM_001329.4	Yes
2	GNB2L1/NM_006098.5	Yes	39	RPL36/NM_033643.3	Yes
3	PSMD3/NM_002809.4	Yes	40	CYC1/NM_001916.5	Yes
4	TAF-I/NM_001122821.2	Yes	41	RPS27A/NM_002954.6	Yes
5	SFRS1/NM_006924.5	Yes	42	ZNF358/NM_018083.5	No
6	FAU/NM_001997.5	NO	43	UBTF/NM_014233.4	Yes
7	SSX2IP/NM_001166417.2	Yes	44	ANKRD11/NM_001256182.2	Yes
8	PYCR1/NM_001330523.1	Yes	45	ASS1/NM_000050.4	Yes
9	HARS2/NM_012208.4	No	46	TOP1MT/NM_052963.3	Yes
10	CHD7/NM_017780.4	Yes	47	DDX39B/NM_004640.7	Yes
11	COASY/NM_025233.7	Yes	48	SMARCA4/NM_001128849.3	Yes
12	HMBS/NM_000190.4	Yes	49	NREP/NM_004772.4	Yes
13	JSRP1/NM_144616.4	No	50	PTMA/NM_001099285.2	Yes
14	RAN/NM_006325.5	Yes	51	HLA-A/NM_002116.8	Yes
15	SSSCA1/NM_006396.3	No	52	CPSF3L/NM_001256456.2	No
16	HMGB2/NM_002129.4	Yes	53	CCDC124/NM_138442.4	No
17	BRD7/NM_001173984.3	Yes	54	HDLBP/NM_005336.6	Yes
18	SUV420H2/NM_032701.4	Yes	55	RPL10/NM_006013.5	Yes
19	CDKN2A/NM_000077.5	Yes	56	RPL7A/NM_000972.3	Yes
20	RIOK1/NM_031480.3	Yes	57	HNRNPA1/NM_002136.4	Yes
21	TTC5/NM_138376.3	Yes	58	GNL2/NM_013285.3	Yes
22	GDI2/NM_001494.4	Yes	59	CCDC83/NM_173556.5	Yes
23	NAPRT1/NM_145201.6	Yes	60	GPBP1L1/NM_021639.5	No
24	MRPL12/NM_002949.4	No	61	GCSH/NM_004483.5	No
25	EIF3F/NM_003754.3	No	62	CCDC158/NM_001042784.1	No
26	MCM3/NM_002388.6	Yes	63	ATP5O/NM_001697.3	No
27	STUB1/NM_005861.4	Yes	64	GDI2/NM_001494.4	Yes
28	HSPA4/NM_002154.4	Yes	65	IQSEC1/NM_001134382.3	Yes
29	NOL4/NM_003787.5	Yes	66	TUBB2/NM_001069.3	Yes
30	PUF60/NM_078480.3	Yes	67	CCDC17/NM_001114938.3	No
31	RPL9/NM_000661.5	Yes	68	ZNF532/NM_018181.6	Yes
32	CDK11A/NM_024011.4	Yes	69	KIF20B/NM_001284259.2	Yes
33	GOSR1/NM_004871.3	Yes	70	TMEM9/NM_016456.5	Yes
34	NME1/NM_198175.1	Yes	71	KIF27/NM_017576.4	No
35	XLS/NM_001329678.2	Yes	72	CLIC4/NM_013943.3	Yes
36	HMGN3/NM_004242.4	No	73	ODC1/NM_002539.3	Yes
37	ACTG1/NM_001199954.3	Yes	74	ST13/NM_003932.5	Yes

^a^ KP-SCLC-1 to -58 and KP-SCLC-59 to -74 were discovered from NCI-H889 cDNA library source and from testis cDNA library source, respectively. ^b^ Analysis of EMBASE database was used to determine whether each gene has at least one published article related to cancer.

**Table 2:** Summary of anti NOL4 antibodies in sera from SCLC patients and healthy donors.

Serum Number	Serum Source ^a^	SeroreacTivity ^b^	Serum Number	Serum Source	Seroreactivity
N1	Healthy donor	N	S13	ED-SCLC	P
N2	Healthy donor	N	S14	ED-SCLC	P
N3	Healthy donor	P	S15	ED-SCLC	P
N4	Healthy donor	P	S16	ED-SCLC	P
N5	Healthy donor	P	S17	ED-SCLC	P
N6	Healthy donor	N	S18	ED-SCLC	N
N7	Healthy donor	N	S19	ED-SCLC	P
N8	Healthy donor	P	S20	ED-SCLC	P
N9	Healthy donor	P	S21	ED-SCLC	P
N10	Healthy donor	P	S22	ED-SCLC	P
N11	Healthy donor	N	S23	ED-SCLC	P
N12	Healthy donor	P	S24	ED-SCLC	P
N13	Healthy donor	N	S25	ED-SCLC	P
N14	Healthy donor	P	S26	ED-SCLC	P
N15	Healthy donor	P	S27	ED-SCLC	P
N16	Healthy donor	P	S28	ED-SCLC	P
N17	Healthy donor	N	S29	ED-SCLC	P
N18	Healthy donor	P	S30	ED-SCLC	P
N19	Healthy donor	P	S31	ED-SCLC	P
N20	Healthy donor	P	S32	LD-SCLC	P
S1	ED-SCLC	N	S33	LD-SCLC	P
S2	ED-SCLC	N	S34	LD-SCLC	P
S3	ED-SCLC	N	S35	LD-SCLC	P
S4	ED-SCLC	N	S36	LD-SCLC	P
S5	ED-SCLC	P	S37	LD-SCLC	N
S6	ED-SCLC	P	S38	LD-SCLC	P
S7	ED-SCLC	P	S39	LD-SCLC	P
S8	ED-SCLC	N	S40	LD-SCLC	N
S9	ED-SCLC	P	S41	LD-SCLC	N
S10	ED-SCLC	N	S42	LD-SCLC	P
S11	ED-SCLC	P	S43	LD-SCLC	P
S12	ED-SCLC	P	S44	LD-SCLC	N

^a^ ED; extensive disease, LD; limited disease, ^b^ P; positive, N; negative.

## Data Availability

Publicly available datasets were analyzed in this study. This data can be found here: GeneCards database (http://bioinfo.weizmann.ac.il/cards-bin/) (accessed on 15 November 2020) and EMBASE (https://www.embase.com/) (accessed on 15 November 2020).

## Supplementary Materials

The following are available online at https://www.mdpi.com/article/10.3390/curroncol28030179/s1, Figure S1: The raw image of western blot film of Table 2.
