# Supplementary material for: Cancer Testis Antigen, NOL4, Is an Immunogenic Antigen Specifically Expressed in Small-Cell Lung Cancer
Source: Curr Oncol. 2021 May 20;28(3):1927–37. doi: 10.3390/curroncol28030179 (PMC8161805; doi:10.3390/curroncol28030179)
Supplement: Supplementary file 1 [file curroncol-28-00179-s001.zip › curroncol-1168059-supplementary.pdf]

Supplementary Materials:

Cancer Testis Antigen, NOL4, Is an Immunogenic Antigen Specifically Expressed in Small Cell Lung Cancer

Ye-Rin Kim, Ki-Uk Kim, Jung-Hee Lee, Deok-Won Kim, Jae-Heun Chung, Yeong-Dae Kim, Dong-Hoon Shin, Min-Ki Lee, Yong-Il Shin and Sang-Yull Lee

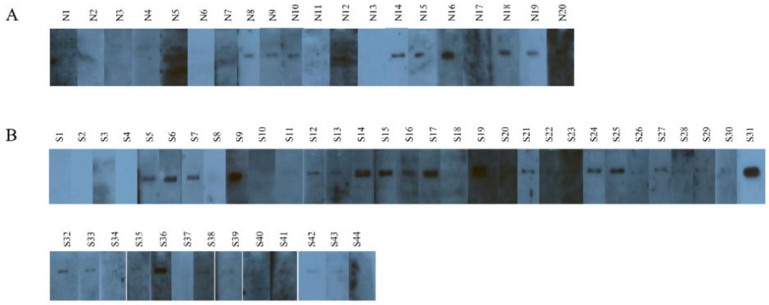

Figure S1. The raw image of western blot film of Table 2.
